# Quantification of viral infection dynamics in animal experiments

**DOI:** 10.3389/fmicb.2013.00264

**Published:** 2013-09-10

**Authors:** Shingo Iwami, Yoshiki Koizumi, Hiroki Ikeda, Yusuke Kakizoe

**Affiliations:** ^1^Department of Biology, Faculty of Sciences, Kyushu UniversityFukuoka, Japan; ^2^School of Medicine, College of Medical, Pharmaceutical and Health Sciences, Kanazawa UniversityKanazawa, Ishikawa, Japan

**Keywords:** virus infection, mathematical modeling, computer simulation, animal experiment, quantification

## Abstract

Analyzing the time-course of several viral infections using mathematical models based on experimental data can provide important quantitative insights regarding infection dynamics. Over the past decade, the importance and significance of mathematical modeling has been gaining recognition among virologists. In the near future, many animal models of human-specific infections and experimental data from high-throughput techniques will become available. This will provide us with the opportunity to develop new quantitative approaches, combining experimental and mathematical analyses. In this paper, we review the various quantitative analyses of viral infections and discuss their possible applications.

## INTRODUCTION

Based on a decline in the viral load of human immunodeficiency virus type-1 (HIV-1) patients following the initiation of antiviral therapy, the turnover of HIV infected cells *in vivo* was estimated through mathematical modeling (Ho et al., 1995; Wei et al., 1995). Starting with these landmark papers, mathematical modeling has evolved into an important tool in modern virology. Developing a quantitative understanding of virus infection dynamics is useful for determining the pathogenesis and transmissibility of viruses, predicting the course of disease, and evaluating the effects of antiviral therapy in HIV (Perelson, 2002; Simon and Ho, 2003; Rong and Perelson, 2009), hepatitis B/C virus (HBV/HCV; Dahari et al., 2008, 2011; Rong and Perelson, 2010; Chatterjee et al., 2012) and influenza virus infection (Beauchemin and Handel, 2011; Murillo et al., 2013). The importance and significance of mathematical modeling work is slowly being recognized by virologists. In addition, in recent years, data from animal experiments have been analyzed using mathematical models (Igarashi et al., 1999; Chen et al., 2007; Dahari and Perelson, 2007; Dinoso et al., 2009; Klatt et al., 2010; Miao et al., 2010; Wong et al., 2010; Graw et al., 2011; Horiike et al., 2012; Pinilla et al., 2012; Oue et al., 2013). A synergistic approach, combining animal experiments and mathematical models, has strong potential applications for researching various viral infections. For example, to determine certain aspects of virus infection, such as sites of infection, target cells (Dinoso et al., 2009; Horiike et al., 2012), and viral gene functions (Sato et al., 2010, 2012; Pinilla et al., 2012), designing an animal experiment and estimating numerous parameters with a mathematical model are useful and important. In the future, to understand the pathophysiology of untreatable or (re-)emerging virus infections, and to effectively develop therapeutic strategies against these viruses, we need to establish a platform involving quantitative analyses that are based on data from animal experiments (Perelson, 2002; Simon and Ho, 2003; Dahari et al., 2008, 2011; Rong and Perelson, 2009, 2010; Beauchemin and Handel, 2011; Chatterjee et al., 2012; Murillo et al., 2013). In this paper, we briefly review a history of quantitative approaches to virology and discuss the possible applications of these in combination with animal experiments.

## QUANTIFICATION OF VIRUS INFECTION DYNAMICS

Virological research has typically been conducted with a small number of experiments. For example, in order to investigate the fitness of virus strains, one typically measured viral loads (e.g., the amount of viral protein and viral infectivity) at a few times during infection and determined whether one strain produces significantly more virus than the other. However, the entire time-course of an infection reflects complex processes involving interactions between viruses, target cells, and infected cells. Therefore, viral load detection at one time point ignores the complexity of the aforementioned processes during an entire infection (Iwami et al., 2012b). It would be useful to translate virus infection quantitatively into the parameters identifying the multi-composed kinetics of viral infection from time-course data (Perelson, 2002; Simon and Ho, 2003; Dahari et al., 2008, 2011; Rong and Perelson, 2009, 2010; Beauchemin and Handel, 2011; Chatterjee et al., 2012; Murillo et al., 2013). Mathematical modeling of the entire time-course of infection would allow us to estimate several parameters underlying the kinetics of virus infection, including burst size and basic reproductive number (Nowak and May, 2000). These parameters cannot be directly obtained through experimental and clinical studies.

### HUMAN IMMUNODEFICIENCY VIRUS AND SIMIAN IMMUNODEFICIENCY VIRUS

On average it takes about, 10 years for an HIV infection to possibly progress to acquired immunodeficiency syndrome (AIDS; Richman, 2001). Because of this slow disease progression, HIV is classified as a slowly replicating virus (Coffin, 1995; Richman, 2001). Several studies have indicated that slow disease progression is not due to inactive viral replication, but is a result of aggressive viral replication and its clearance (Coffin, 1995; Ho et al., 1995; Wei et al., 1995; Perelson et al., 1996). Interestingly, these results were based on mathematical analyses of clinical data. Estimating the decline in viral load of patients following the initiation of antiviral therapy (or plasma removal by apheresis technique; Ramratnam et al., 1999) shows us that HIV is cleared from patients at a rapid rate, with a half-life of around 6 h (Ho et al., 1995; Wei et al., 1995; Perelson et al., 1996). This estimation of rapid virus turnover implies that HIV resistance to any single drug could quickly emerge, highlighting the importance of combination therapies as they reduce the chances of drug resistance developing (Coffin, 1995; Ho et al., 1995; Wei et al., 1995; Perelson et al., 1996; Perelson and Nelson, 1999).

The success of mathematical modeling, especially with respect to HIV infection dynamics has led to the development of a field called “viral dynamics” (Nowak and May, 2000; Perelson, 2002; Simon and Ho, 2003; Rong and Perelson, 2009) and has provided us with further quantitatively novel insights. In 1997, combinations of three antiretroviral drugs successfully reduced plasma HIV levels to below the limit of detection in clinical assays (50 copies of HIV RNA/ml; Perelson et al., 1997; Eisele and Siliciano, 2012). This approach, known as highly active antiretroviral therapy (HAART), is currently the primary choice of therapeutic intervention for HIV-1 infected patients, and dramatically decreases mortality associated with HIV-1 infection (Richman, 2001; Trono et al., 2010; Eisele and Siliciano, 2012). After the initiation of HAART, the viral load decays with an initially rapid and exponential decline, followed by a slower exponential decline (Perelson et al., 1997; Perelson, 2002; Murray et al., 2007; Keele et al., 2008; Palmer et al., 2008; Rong and Perelson, 2010). Modeling the effects of drug therapy allowed for the quantitation of virus clearance rates (Ho et al., 1995; Wei et al., 1995; Perelson et al., 1996), and the death rate of several cell types (e.g., the productively infected CD4^+^ T cells; Perelson et al., 1996, 1997; Markowitz et al., 2003), the productively long-lived infected cells (Perelson et al., 1997; Palmer et al., 2008), latently infected cells (Perelson et al., 1997; Zhang et al., 1999; Havlir et al., 2003; Palmer et al., 2008 and so on). This modeling also estimates the period of infectiousness for follicular dendritic cell-trapping viruses (Hlavacek et al., 2000), and assists with designing an optimal therapy (Murray et al., 2007; Rosenbloom et al., 2012). A simple assessment of HIV RNA data yields certain information, but mathematical approaches allow for the extraction of much more information from raw data. Some excellent reviews regarding the quantitation of HIV dynamics and its importance have been reported (Perelson and Nelson, 1999; Nowak and May, 2000; Perelson, 2002; Simon and Ho, 2003; Rong and Perelson, 2009).

Potent HAART effectively suppresses *de novo* replication of HIV but fails to eradicate an HIV-1 infection. Recent studies have revealed that HIV RNA persists over several years in most infected patients on suppressive HAART (Havlir et al., 2003; Palmer et al., 2008). Furthermore, virus loads rapidly rebound to pretreatment levels after discontinuation of HAART (Chun et al., 1999; Imamichi et al., 2001). These observations suggest the persistence of viral reservoirs during combination antiviral therapy. To completely cure HIV-1 infection, it is essential to identify these viral reservoirs and to eradicate them (Richman, 2001; Trono et al., 2010; Eisele and Siliciano, 2012). In addition to existing studies looking at peripheral blood (Chun et al., 1999; Imamichi et al., 2001; Sharkey et al., 2011), systemic analysis is required to elucidate the mechanisms underlying rebound of plasma viremia upon discontinuation of HAART. Because it is unethical to collect various tissues from patients, or to deplete certain cell populations in patients for analysis, the simian immunodeficiency virus (SIV)/macaque model (which has been useful in understanding HIV-1 infection) with HAART is suitable for investigating poorly understood aspects of HIV-1 infection (Dinoso et al., 2009; North et al., 2010; Horiike et al., 2012; Oue et al., 2013). Using the SIV/macaque model, for example, it has been recently reported that the cytotoxic effects of CD8^+^ T cells on virus-infected cells during HAART is limited despite suppression of viral load *in vivo* (Klatt et al., 2010; Wong et al., 2010). Similarly, analyzing the levels of viral RNA in plasma and infected cells (e.g., macrophage and resting memory CD4^+^ T cells) of certain tissues such as lung and lymph nodes in SIV-infected macaques using mathematical models might reveal the precise dynamics of the viral reservoir, and provide several valuable clues for HIV eradication in patients on HAART.

### HEPATITIS B VIRUS AND HEPATITIS C VIRUS

The theoretical framework for quantifying HIV infection has also been applied to understand the dynamics of HBV (Nowak et al., 1996; Lewin et al., 2001; Murray et al., 2006; Dahari et al., 2009b) and HCV (Neumann et al., 1998; Dixit et al., 2004; Guedj and Perelson, 2011; Guedj et al., 2012) infections during antiviral therapy. These approaches have estimated the key parameters of the viral life cycle such as the rate of virus production and clearance and the death rate of infected cells, that explained the mechanism of action of antiviral drugs such as interferon, ribavirin, and protease inhibitor against HCV (Neumann et al., 1998; Dixit et al., 2004; Guedj and Perelson, 2011; Guedj et al., 2012) and reverse transcriptase inhibitor against HBV (Nowak et al., 1996; Lewin et al., 2001; Murray et al., 2006; Dahari et al., 2009b). These analyses have mainly focused on extracellular viral dynamics based on clinical studies, while several researchers have investigated the intracellular replication of HCV (Dahari et al., 2007, 2009a; McLean et al., 2010; Nakabayashi, 2012) and HBV (Nakabayashi and Sasaki, 2011) based on experimentally established HBV/HCV cell culture system. These studies have provided novel insights into the detailed dynamics of intracellular HBV/HCV replication, and revealed some important processes of the HCV life cycle such as the subcellular localization of HCV RNA to the replication complex for RNA replication and viral assembly. The above findings are helpful in understanding HCV turnover and determining new drug targets with fewer side effects. A number of reviews have been published detailing the mathematical modeling of HCV infection (Dahari et al., 2008; Guedj et al., 2010; Rong and Perelson, 2010; Guedj and Perelson, 2011; Chatterjee et al., 2012).

Although mathematical models were successfully used to understand the viral dynamics of HBV/HCV during antiviral therapy, these models considered only one level of extracellular or intracellular viral replication. Recently, for HCV infection, several researchers have developed a new mathematical model, known as the multi-scale model, that combines extracellular virus infection dynamics with the key features of intracellular viral replication (Guedj and Neumann, 2010; Guedj et al., 2013; Rong et al., 2013). This model incorporates two different time scales: one is for viral replication within a cell, and the other is for free viral infection among cells. Using this model in conjunction with clinical trials, it is possible to verify the mechanism of action of direct-acting antiviral agents (DAAs) that target specific viral proteins in a cell. Estimating the effectiveness of DAAs using multi-scale model takes into account intracellular viral dynamics (Guedj et al., 2013; Rong et al., 2013). Additionally, the multi-scale model has the potential to describe the emergence of viral drug resistance against DAAs at an intracellular and extracellular level (Guedj and Neumann, 2010). In the era of developing DAAs, multi-scale models could provide a new theoretical framework that combines findings from several studies of intra- and extracellular viral dynamics; this can be applied to HBV, HIV (Althaus and De Boer, 2010), and influenza virus (Murillo et al., 2013).

Using animal and cell culture systems with mathematical models paves the way to investigate new vaccines against HCV. It would also assist with understanding the mechanisms of antiviral drug therapy. HBV vaccines are available, but there is no effective vaccine against HCV infection. The development of an effective HCV vaccine has been hampered by the high mutation rate of viral proteins, the genetic diversity of HCV, and the lack of usable small animal models for HCV infection (Houghton and Abrignani, 2005; Klenerman and Gupta, 2012). Recently, a uPA-TG/severe combined immunodeficiency (SCID) mouse model for HCV infection has been developed (Mercer et al., 2001). Although an authentic immune response against HCV does not occur in these models (and therefore cannot be directly suitable for vaccine studies), mathematical modeling could compensate for the lack of information regarding key processes of HCV immune interactions and promote further development of small animal models of HCV. These and other animal models could be alternatives to chimpanzees for investigating the effects of candidates drugs and vaccines against HCV (Bukh, 2012; chimpanzees are endangered species and now cannot be used for animal experiments). On the other hand, mathematical modeling of the immune response against HBV in patients has estimated the contribution of the host response for viral clearance (Ciupe et al., 2007a,b), and the optimal vaccination schedule (Gesemann and Scheiermann, 1995; Wilson et al., 2007). Taken together, the combination of a small animal model and mathematical modeling can overcome the ethical and financial limitations of clinical trials and help develop new effective therapies against HBV and HCV.

### INFLUENZA VIRUS

In epidemiology, many mathematical models have been developed and been used to determine the dynamics of influenza virus infections on the population level (Anderson, 1991; Ferguson et al., 2006; Hatchett et al., 2007; Beauchemin and Handel, 2011; Murillo et al., 2013). A small number of models have also been generated to describe influenza virus infections at the host level (Baccam et al., 2006; Miao et al., 2010; Dobrovolny et al., 2011; Pinilla et al., 2012; and at the individual cell level; Hatada et al., 1989; Heldt et al., 2012). The purpose of these models is to describe the time-course of influenza virus infections as accurately as possible. This allows for the calculation of the half-life of infected cells, the number of virus particles released per infected cell (i.e., the burst size), and the number of infected cells produced per infected cell (i.e., the basic reproductive number; Baccam et al., 2006; Beauchemin et al., 2008; Mitchell et al., 2011; Pinilla et al., 2012). This information has been used to understand the severity and duration of infections (Bocharov and Romanyukha, 1994; Hancioglu et al., 2007; Canini and Carrat, 2011), and has provided us with an optimal antiviral therapy (Baccam et al., 2006; Handel et al., 2007; Dobrovolny et al., 2011; Perelson et al., 2012).

Although influenza viruses have been studied extensively *in vivo*, it is difficult to determine the exact date of infection, influenza virus loads prior to a peak, and pre-hemagglutination inhibition antibody titers. All these factors are crucial in quantifying virus infection dynamics (Murphy et al., 1980; Carrat et al., 2008). Experimental infection of healthy volunteers with influenza viruses provides a unique opportunity to elucidate the dynamics of natural influenza infections. The first mathematical model proposed to describe the dynamics of influenza infections, using influenza A/Hong Kong/123/77 (H1N1), was conducted in 2006 (Baccam et al., 2006). This simple mathematical model revealed several important and novel quantities corresponding to biological processes of influenza virus infection (Mohler et al., 2005; Baccam et al., 2006; Schulze-Horsel et al., 2009; Smith et al., 2010; Beauchemin and Handel, 2011; Holder and Beauchemin, 2011; Murillo et al., 2013). For example, if a basic reproductive number (*R*_0_) was obtained, the critical inhibition rate (1 - 1/*R*_0_) could be estimated for protection against virus infection (Anderson, 1991; Iwami et al., 2012a,b). This implies that reducing viral growth with antiviral interventions, such as vaccines or drugs, could prevent viral spread *in vivo*. More biologically realistic mathematical models incorporating the eclipse phase of infected cells (Holder and Beauchemin, 2011; Pinilla et al., 2012), innate or adaptive immune responses (Bocharov and Romanyukha, 1994; Hancioglu et al., 2007; Handel et al., 2007; Miao et al., 2010; Canini and Carrat, 2011), and several distributed delays for each biological process (Holder and Beauchemin, 2011; e.g., a time from virus entry to progeny virus producing) have been developed. Furthermore, using a mathematical model, a relationship between virus and symptom dynamics during influenza infections has been described recently (Canini and Carrat, 2011). Reviews regarding quantitation of influenza virus dynamics and its importance have been published previously (Beauchemin and Handel, 2011; Murillo et al., 2013).

These quantitative analyses of influenza viruses have yielded useful insights (Bocharov and Romanyukha, 1994; Mohler et al., 2005; Baccam et al., 2006; Hancioglu et al., 2007; Handel et al., 2007; Schulze-Horsel et al., 2009; Miao et al., 2010; Smith et al., 2010; Canini and Carrat, 2011; Dobrovolny et al., 2011; Holder and Beauchemin, 2011; Perelson et al., 2012; Pinilla et al., 2012; Murillo et al., 2013). The development of reliable within-host models is critical in improving epidemiological models because the dynamics of viral shedding and symptoms following influenza virus infection are key factors (Ferguson et al., 2006; Hatchett et al., 2007; Murillo et al., 2013). However, a major difficulty, over-parameterization (Beauchemin and Handel, 2011), arises when only viral load data are available, especially in human volunteer studies. One possible approach to overcome this limitation is to conduct animal experiments using rhesus macaques (Watanabe et al., 2011), ferrets (Kiso et al., 2010), and mice (Imai et al., 2008; Martin and Wurfel, 2008; Morita et al., 2013), and to measure the time-course data for these analyses (Miao et al., 2010; Pinilla et al., 2012). It is possible that the number of uninfected and infected cells from the lung or respiratory tract can be measured. Although it is still currently not feasible to obtain sufficient time-course data during the acute phase of infections, we have recently developed a novel but simple mathematical model to robustly estimate virus replication rates (Ikeda et al., unpublished). In this model, a relatively few time-course data of both the number of uninfected cells and viral load is required. A new model and diverse data will promote knowledge of influenza virus infection dynamics, which is important for future research.

### OTHER VIRUSES

Mathematical models have also been applied for understanding the dynamics of other virus infections. For example, during acute lymphocytic choriomeningitis virus (LCMV, which is a common infection of rodents and is best known for its application in immunological studies) infection, the dynamics of the specific CD8^+^ T-cell response such as proliferation and apoptosis rate was estimated in infected mice (De Boer et al., 2001). Quantitative analyses suggested that the specific CD8^+^ T-cell response is controlled via the number of CD8^+^ T-cells, rather than their individual function during persistent LCMV infection compared with those in acute LCMV infections (in fact, the immune response with a high killing effect is necessary to clear the LCMV infection; Graw et al., 2011). On the other hand, modeling and fitting data from patients revealed that the doubling time of cytomegaloviruses (CMV, which is assumed to cause asymptomatic infection in normal hosts) in human hosts is around 1 day, similar to that for HIV (Emery et al., 1999; Perelson, 2002). More recently, it has been revealed that viral productivity and transmissibility, but not cytotoxicity, differ among Enterovirus 71 (EV71, which is the causative agent of hand-foot-and-mouth disease and can trigger neurological disorders) strains in cell culture and could be associated with their epidemiological backgrounds (Fukuhara et al., 2013). Animal experiments using monkeys and mice are available to investigate the pathogenesis and symptoms of these and numerous other viruses (Farrell et al., 1997; De Boer et al., 2001; Arita et al., 2007, 2008; Graw et al., 2011; Sato et al., 2011). We have a chance to establish a platform that will allow for quantitative understanding of various virus infections based on animal experiments. Accumulation of knowledge regarding viral dynamics should be useful in understanding untreatable or (re-)emerging virus infections.

## CONCLUSION

**FIGURE 1:**
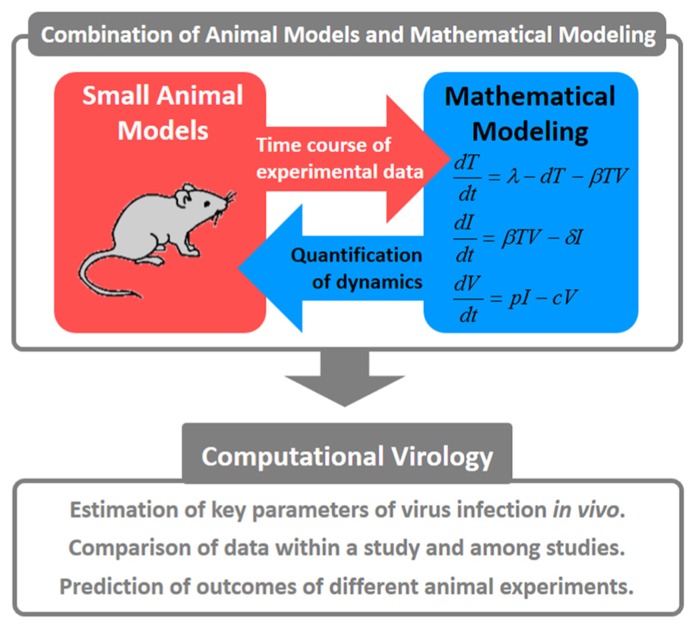
**Concepts for combining animal models with mathematical modeling.** These quantitative approaches are referred to as “computational virology.” This has the potential to provide novel insights into viral pathogenesis, the development of antiviral drugs, and the establishment of effective therapies against viral infections.

Studies of virus infection dynamics have significantly contributed to our understanding of many diseases. Merging animal experiment results with mathematical models is a desirable direction for virology research (**Figure 1**). In particular, quantifying viral dynamics in “humanized mice” (Shultz et al., 2007; Sato and Koyanagi, 2011), which are the most practical and relevant model available, will provide us with novel insights. Using humanized mice as models of specific human viral infections (Sato et al., 2010, 2011, 2012) or human diseases (Ishikawa et al., 2007), we were able to investigate mechanisms of disease symptoms (e.g., a relation between function of regulatory T cells and depletion of CD4^+^ T cells in HIV-1 infection), and the potency/mechanism of action for drug/host factors (e.g., an effect of anticancer drug in human T cell leukemia virus type-1 infection) based on virus infection dynamics. Mathematical models can be used to explore a complicated dynamical system of virus infection. Estimation of key parameters during a virus infection provides us with many details regarding the infection. If we can obtain these estimated parameters and calculate burst size and basic reproductive numbers, we could easily compare the dynamics of various viral infections. Qualitative data (in fact, most experiments are not designed from a quantitative point of view) are difficult to understand and compare with results from other studies. Based on the theoretical analysis of experimental data, we are able to determine optimum frequencies of sampling for the highest quality data possible. Once we establish a mathematical model and reasonably fit that model to experimental data, we can predict outcomes of animal experiments under different conditions and determine factors that control several phenomena during virus infection (e.g., peak of viral load and mode of virus spread) through simulations. Further associations between animal experiments and mathematical models are required to overcome untreatable diseases.

## Conflict of Interest Statement

The authors declare that the research was conducted in the absence of any commercial or financial relationships that could be construed as a potential conflict of interest.
